# 1-{(*E*)-[(2*E*)-3-(4-Meth­oxy­phen­yl)-1-phenyl­prop-2-en-1-yl­idene]amino}-3-phenyl­urea: crystal structure and Hirshfeld surface analysis

**DOI:** 10.1107/S2056989017014128

**Published:** 2017-10-06

**Authors:** Ming Yueh Tan, Karen A. Crouse, Thahira B. S. A. Ravoof, Mukesh M. Jotani, Edward R. T. Tiekink

**Affiliations:** a Department of Physical Science, Faculty of Applied Sciences, Tunku Abdul Rahman University College, 50932 Setapak, Kuala Lumpur, Malaysia; b Department of Chemistry, Faculty of Science, Universiti Putra Malaysia, 43400 UPM Serdang, Selangor Darul Ehsan, Malaysia; c Department of Chemistry, St. Francis Xavier University, PO Box 5000, Antigonish, NS B2G 2W5, Canada; d Department of Physics, Bhavan’s Sheth R. A. College of Science, Ahmedabad, Gujarat 380 001, India; e Research Centre for Crystalline Materials, School of Science and Technology, Sunway University, 47500 Bandar Sunway, Selangor Darul Ehsan, Malaysia

**Keywords:** crystal structure, urea derivative, hydrogen bonding, Hirshfeld surface analysis

## Abstract

The title compound, C_23_H_21_N_3_O_2_, is constructed about an almost planar disubstituted amino­urea residue (r.m.s. deviation = 0.0201 Å), which features an intra­molecular amine-N—H⋯N(imine) hydrogen bond. In the ‘all-*trans*’ chain connecting this to the terminal meth­oxy­benzene residue, the conformation about each of the imine and ethyl­ene double bonds is *E*. In the crystal, amide-N—H⋯O(carbon­yl) hydrogen bonds connect centrosymmetrically related mol­ecules into dimeric aggregates, which also incorporate ethyl­ene-C—H⋯O(amide) inter­actions. The dimers are linked by amine–phenyl-C—H⋯π(imine–phen­yl) and meth­oxy­benzene-C—H⋯π(amine–phen­yl) inter­actions to generate a three-dimensional network. The importance of C—H⋯π inter­actions in the mol­ecular packing is reflected in the relatively high contributions made by C⋯H/H⋯C contacts to the Hirshfeld surface, *i.e*. 31.6%.

## Abstract 2

An intra­molecular amine-N—H⋯N(imine) hydrogen bond is found in the disubstituted amino­urea residue; the conformations about the imine and ethyl­ene double bonds are *E*. The packing features amide-N—H⋯O(carbon­yl) hydrogen bonds, leading to centrosymmetric aggregates, as well as C—H⋯O and C—H⋯π inter­actions, which significantly influence the packing.

## Chemical context   

Chalcones are natural or synthetic compounds comprising an open-chain flavonoid structure in which the two aromatic rings are connected *via* a three-carbon-atom α,β-unsaturated carbonyl system. These compounds have attracted much attention due to their diverse pharmacological and biological activities (Gaonkar & Vignesh, 2017 ▸), including their anti-cancer (Mahapatra *et al.*, 2015 ▸), anti-malarial (Syahri *et al.*, 2017 ▸), anti-inflammatory (Li *et al.*, 2017 ▸), anti-microbial (Kumar *et al.*, 2017 ▸), xanthine oxidase inhibitory (Xie *et al.*, 2017 ▸) and aldol reductase inhibitory (Zhuang *et al.*, 2017 ▸) properties. The present work is part of an on-going project on the synthesis of chalcone-derived Schiff bases, their ultilization in the synthesis of new transition metal complexes and their investigation as anti-proliferative and anti-bacterial agents. In this context, crystal-structure determinations of a chalcone-derived thio­semicarbazone and a zinc complex have been published (Tan *et al.*, 2015 ▸, 2017 ▸).

In this contribution, a chalcone residue has been incorporated into a semicarbazide skeleton to form the title chalcone­semicarbazone, (I). While chalconesemicarbazone derivatives have shown potential anti-convulsant (Sharma *et al.*, 2014 ▸), anti-inflammatory (Singha *et al.*, 2010 ▸) and anti-oxidant activities (Singhal *et al.*, 2011 ▸), no crystal structures of chalconesemicarbazone derivatives have been published. Herein, the crystal and mol­ecular structures of (I) have been determined and the study augmented by an analysis of the calculated Hirshfeld surfaces.
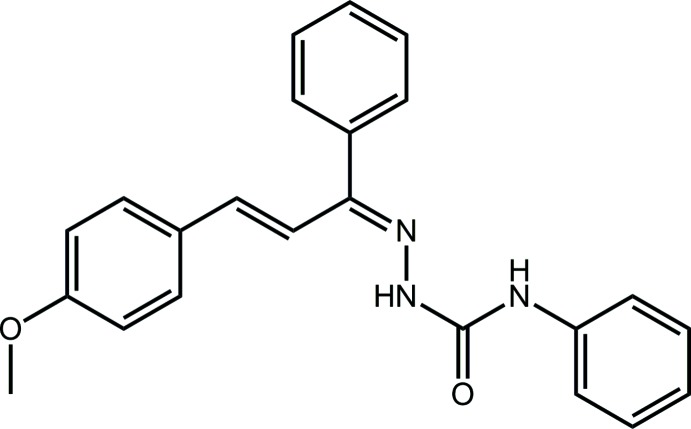


## Structural commentary   

**Figure 1:**
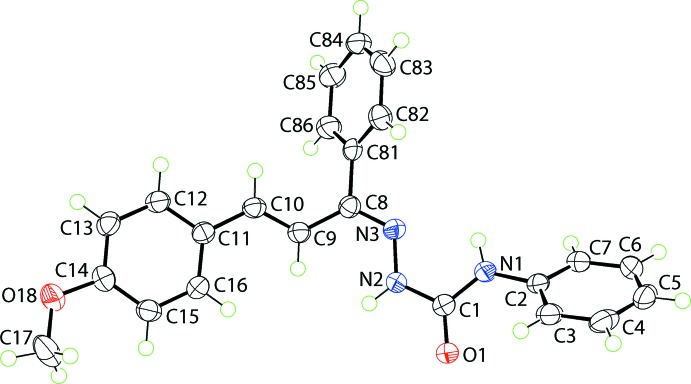
The mol­ecular structure of (I), showing the atom-labelling scheme and displacement ellipsoids at the 70% probability level.

The mol­ecular structure of (I), Fig. 1 ▸, comprises a doubly substituted amino­urea residue which is close to planar (r.m.s. deviation of CN_3_O = 0.0201 Å), owing in part to an intra­molecular amine-N—H⋯N(imine) hydrogen bond, Table 1 ▸. The amine-bound phenyl ring is inclined to the CN_3_O plane, forming a dihedral angle of 46.88 (4)°. The imine/ethyl­ene sequence of bonds, *i.e*. N3=C8—C9=C10—C11, has an all-*trans* conformation but the N3—C8—C9—C10 and C8—C9—C10—C11 torsion angles of 154.62 (12) and −169.19 (11)°, respectively, indicate some twisting in this residue, especially about the C8—C9 bond; the conformation about each of the double bonds is *E*. The imine-bound phenyl ring forms a dihedral angle of 63.30 (7)° with the C_4_N atoms of the imine/ethyl­ene sequence, and the corresponding angle for the terminal meth­oxy­benzene ring is significantly less, at 8.29 (13)°. The meth­oxy group is twisted out of the plane of the ring to which it is connected as seen in the value of the C17—O18—C14—C15 torsion angle of 15.55 (17)°.

## Supra­molecular features   

**Figure 2:**
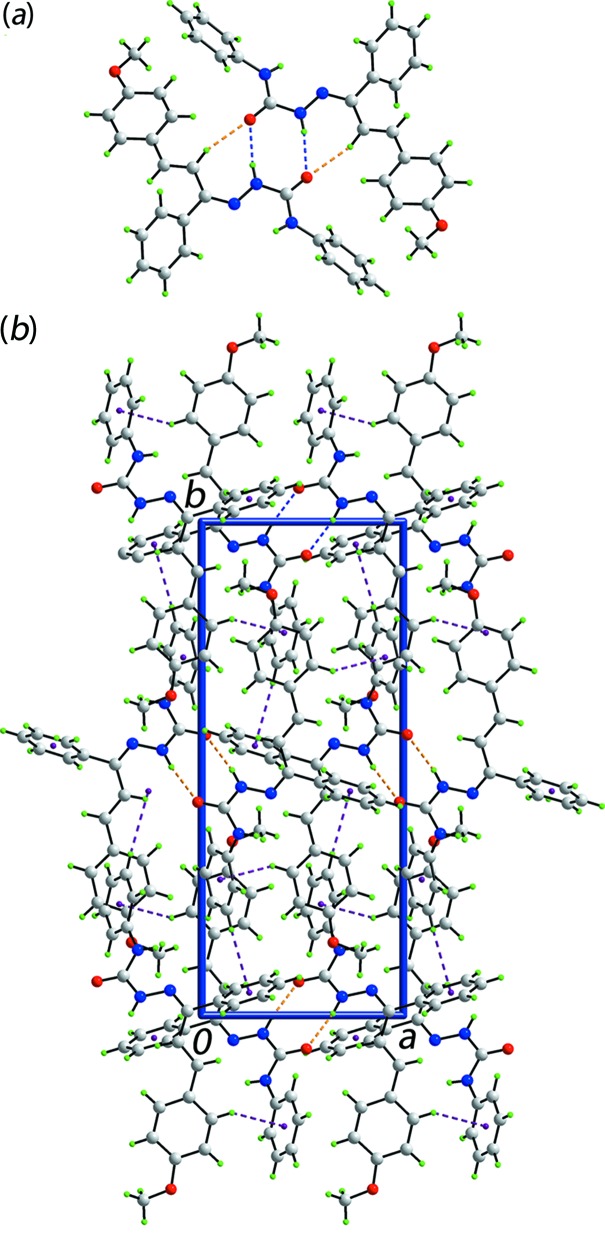
The mol­ecular packing in (I): (*a*) a view of the supra­molecular dimer sustained by amine-N—H⋯O(carbon­yl) hydrogen bonds and supported by ethyl­ene-C—H⋯O(amide) inter­actions, shown as blue and orange dashed lines, respectively, and (*b*) a view of the unit-cell contents shown in projection down the *c* axis. The C—H⋯π inter­actions are shown as purple dashed lines.

The most notable feature of the mol­ecular packing of (I) is the presence of a centrosymmetric, eight-membered amide synthon, {⋯OCNH}_2_, Table 1 ▸. The resultant dimeric aggregate also incorporates two additional ethyl­ene-C—H⋯O(amide) inter­actions, Fig. 2 ▸ *a*, as well as meth­oxy-C—H⋯π(amine-phen­yl) contacts, Table 1 ▸. The aggregates are assembled into a three-dimensional network *via* amine-phenyl-C—H⋯π(imine-phen­yl) and meth­oxy-benzene-C—H⋯π(amine-phen­yl) inter­actions, Fig. 2 ▸ *b*.

## Analysis of the Hirshfeld surface   

**Figure 3:**
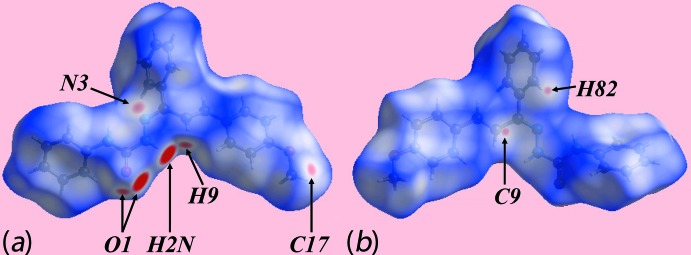
Two views of the Hirshfeld surface for (I) mapped over *d*_norm_ in the ranges (*a*) −0.225 to +1.332 a.u. and (*b*) −0.110 to +1.332 a.u.

**Figure 4:**
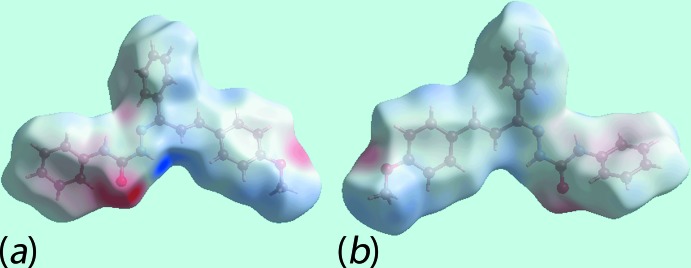
Two views of the Hirshfeld surface for (I) mapped over the electrostatic potential in the range −0.095 to +0.108 a.u. The red and blue regions represent negative and positive electrostatic potentials, respectively.

**Figure 5:**
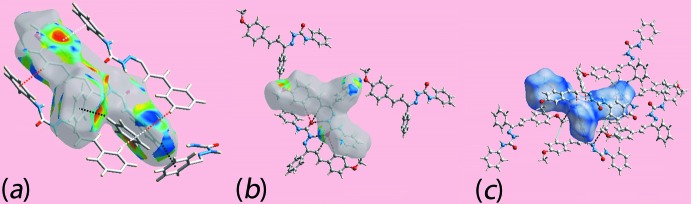
Views of the Hirshfeld surfaces about a reference mol­ecule mapped over (*a*) the shape-index property showing C—H⋯π/π⋯H—C inter­actions involving the C6 atom with the imine–phenyl C81–C86 ring (black dotted lines) and the C12 and C15 atoms with the amine–phenyl C2–C7 rings by red and white dotted lines, respectively, (*b*) the shape-index property about a reference mol­ecule showing short O⋯H/H⋯O contacts by red dotted lines and inter­molecular N—H⋯O and C—H⋯O inter­actions by black dashed lines and (*c*) *d*_norm_ showing short inter­atomic H⋯H, C⋯N/N⋯C and C⋯H/H⋯C contacts by sky-blue, black and red dashed lines, respectively.

The Hirshfeld surface was calculated for (I) in accord with a recent report on a related mol­ecule (Tan *et al.*, 2017 ▸) to provide more detailed information on the relative significance of the various inter­molecular inter­actions. The donors and acceptors of inter­molecular N—H⋯O and C—H⋯O inter­actions in (I) are viewed as the bright-red spots near the ethyl­ene-H9, amide-H2*N* and carbonyl-O1 atoms on the Hirshfeld surface mapped over *d*_norm_ in Fig. 3 ▸ *a*. The appearance of diminutive red spots near the N3 and C17 atoms, Fig. 3 ▸ *a*, and the tiny faint-red spots near the C9 and H82 atoms in Fig. 3 ▸ *b*, indicate the influence of short inter­atomic N3⋯C17 and C9⋯H82 contacts, Table 2 ▸. The donors and acceptors of inter­molecular hydrogen bonds also appear as blue and red regions, respectively, around the participating atoms on the Hirshfeld surface mapped over the calculated electrostatic potential in Fig. 4 ▸. The involvement of the imine-phenyl (C81–C86) and amine-phenyl (C2–C7) rings as acceptors for C—H⋯π inter­actions are also evident through the light-red regions around these rings on the Hirshfeld surfaces in the views of Fig. 4 ▸. Referring to Fig. 5 ▸ *a*, the concave region around the imine-phenyl ring on one side and the biconcave region around the amine-phenyl ring indicate their involvement in one and two C—H⋯π contacts, respectively. The short inter­atomic O⋯H/H⋯O contacts (Table 3 ▸) as well as N—H⋯O and C—H⋯O inter­actions about a reference mol­ecule within shape-index mapped Hirshfeld surface, and the H⋯H, C⋯H/H⋯C and C⋯N/N⋯C contacts within the *d*_norm_-mapped Hirshfeld surface are shown in Fig. 5 ▸ *b* and *c*, respectively.

**Figure 6:**
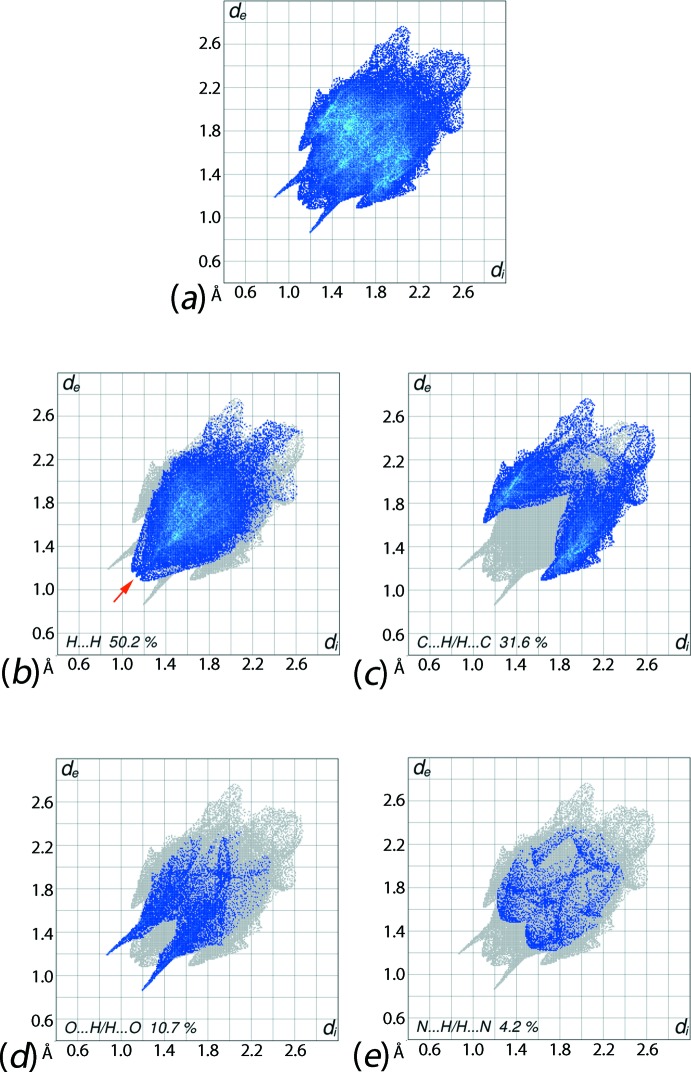
(*a*) The full two-dimensional fingerprint plot for (I) and those delineated into (*b*) H⋯H, (*c*) C⋯H/H⋯C, (*d*) O⋯H/H⋯O and (*e*) N⋯H/H⋯N contacts.

The overall two dimensional fingerprint plot, Fig. 6 ▸ *a*, and those delineated into H⋯H, C⋯H/H⋯C, O⋯H/H⋯O and N⋯H/H⋯N contacts (McKinnon *et al.*, 2007 ▸) are illustrated in Fig. 6 ▸ *b*–*e*, respectively; the relative contributions from different inter­atomic contacts to the Hirshfeld surfaces are summarized in Table 3 ▸. The presence of a small but, distinctive peak at *d*_e_ + *d*_i_ ∼ 2.3 Å in the fingerprint plot delineated into H⋯H contacts, and highlighted by a red arrow in Fig. 6 ▸ *b*, results from the short inter­atomic H⋯H contact between symmetry-related imine-phenyl-H86 atoms, Table 2 ▸, whereas the flanking peaks, at the same *d*_e_ + *d*_i_ ∼ 2.3 Å distance correspond to short inter­atomic H⋯H contacts between meth­oxy­benzene-H12 and meth­oxy-H17*C* atoms, Table 2 ▸.

The C⋯H/H⋯C contacts in the crystal make the second largest contribution, *i.e*. 31.6%, to the Hirshfeld surface of (I), Fig. 6 ▸ *c*, which is due to the presence of a significant number of C—H⋯π inter­actions involving the imine- and amine-phenyl rings, as well as short inter­atomic C⋯H/H⋯C contacts, Table 3 ▸, between the atoms of the meth­oxy-phenyl and imine-phenyl rings, Fig. 5 ▸ *c*. The pair of forceps-like long tips at *d*_e_ + *d*_i_ = 2.1 Å in the fingerprint plot delineated into O⋯H/H⋯O contacts, Fig. 6 ▸ *d*, reflect the presence the N—H⋯O hydrogen bond; the pair of spikes corresponding to the C—H⋯O contacts and the points related to short inter­atomic O⋯H/H⋯O contacts, Table 2 ▸, are merged within the plot. Although the N⋯H/H⋯N contacts have a notable contribution of 4.2% to the Hirshfeld surface, Fig. 6 ▸ *e*, as their inter­atomic distances are greater than their van der Waals separations, they do not make a specific contribution to the mol­ecular packing. The participation of the methyl-C17 atom in two close inter­atomic contacts, Table 2 ▸, brings into close proximity the methyl-C17 and imine-N3 atoms, Table 2 ▸, but these are inter­spersed by the H17*A* and H17*B* atoms so are not surface contacts. Finally, the small contributions from other inter­atomic contacts summarized in Table 3 ▸ have a negligible effect on the structure.

## Database survey   

The title compound was prepared from the de­hydrogenation reaction of 4-phenyl­semicarbazide and 4-meth­oxy­chalcone. A search of the Cambridge Structural Database (Groom *et al.*, 2016 ▸) revealed no direct precedents for this type of mol­ecule. The most closely related structure is one where the ethyl­ene bond is incorporated within a five-membered pyrazolone ring (Chai *et al.*, 2005 ▸). Here, the intra­molecular amine-N—H⋯N(imine) hydrogen bond persists in each of the two independent mol­ecules comprising the asymmetric unit, as do the *E*-conformations about the two analogous double bonds in the mol­ecule. However, there is considerable twisting about the equivalent bonds to C8—C9 in (I), *i.e*. the N—C—C—C torsion angles are 130.3 (6) and 136.0 (6)°, *cf*. 154.62 (12)° in (I), an observation attributed to the need to reduce steric hindrance between the rings in the mol­ecules.

## Synthesis and crystallization   

Analytical grade reagents were used as procured without further purification. 4-Phenyl­semicarbazide (1.51 g, 0.01 mol) and 4-meth­oxy­chalcone (2.38 g, 0.01 mol) were dissolved separately in hot absolute ethanol (30 ml) and mixed with stirring. A few drops of concentrated hydro­chloric acid were added as a catalyst. The reaction mixture was heated and stirred for about 20 min., then stirred for a further 30 min. at room temperature. The resulting yellow precipitate was filtered, washed with cold ethanol and dried *in vacuo*; yield: 75%. Single crystals were grown at room temperature from the slow evaporation of mixed ethanol and aceto­nitrile solvents (1:1 *v*/*v*; 20 ml), m.p. 407 K. IR (cm^−1^): 3336 ν(N—H), 1679 ν(C=O), 1526 ν(C=N), 1242 ν(C—N), 1025 ν(C=S). MS (*m*/*z*): 371.25 [*M*+1]^+^.

## Refinement   

Crystal data, data collection and structure refinement details are summarized in Table 4 ▸. The carbon-bound H atoms were placed in calculated positions (C—H = 0.95–0.98 Å) and were included in the refinement in the riding-model approximation, with *U*_iso_(H) set to 1.2–1.5*U*_eq_(C). The nitro­gen-bound H atoms were located in a difference-Fourier map but were refined with a distance restraint of N—H = 0.88±0.01 Å, and with *U*_iso_(H) set to 1.2*U*_eq_(N).

## Supplementary Material

Crystal structure: contains datablock(s) . DOI: 10.1107/S2056989017014128/hb7709sup1.cif

Structure factors: contains datablock(s) I. DOI: 10.1107/S2056989017014128/hb7709Isup2.hkl

Supporting information file. DOI: 10.1107/S2056989017014128/hb7709Isup3.cml

CCDC reference: 1577439

Additional supporting information:  crystallographic information; 3D view; checkCIF report

## Figures and Tables

**Table 1:** Hydrogen-bond geometry (Å, °) *Cg*1 and *Cg*2 are the centroids of the C2–C7 and C81–C86 rings, respectively.

*D*—H⋯*A*	*D*—H	H⋯*A*	*D*⋯*A*	*D*—H⋯*A*
N1—H1*N*⋯N3	0.87 (1)	2.18 (2)	2.6029 (15)	110 (1)
N2—H2*N*⋯O1^i^	0.88 (1)	2.05 (1)	2.9184 (14)	171 (1)
C9—H9⋯O1^i^	0.95	2.39	3.2913 (15)	159
C15—H15⋯*Cg*1^i^	0.95	2.88	3.5125 (14)	125
C6—H6⋯*Cg*2^ii^	0.95	2.92	3.8296 (14)	161
C12—H12⋯*Cg*1^iii^	0.95	2.75	3.4715 (14)	133

Symmetry codes: (i) 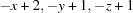; (ii) 
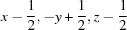
; (iii) 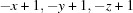.

**Table 2:** Summary of short inter­atomic contacts (Å) in (I)

Contact	Distance	Symmetry operation
C17⋯N3	3.1147 (18)	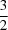 − *x*, − 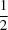 + *y*, 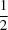 − *z*
C9⋯H82	2.72	1 − *x*, 1 − *y*, 1 − *z*
H86⋯H86	2.26	1 − *x*, 1 − *y*, −*z*
H12⋯H17*C*	2.26	− 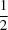 + *x*, 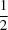 − *y*, − 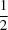 + *z*
O1⋯H16	2.61	2 − *x*, 1 − *y*, 1 − *z*
O18⋯H84	2.67	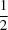 − *x*, − 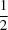 + *y*, 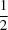 − *z*
C8⋯H82	2.87	1 − *x*, 1 − *y*, 1 − *z*
C12⋯H17*C*	2.79	− 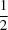 + *x*, 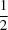 − *y*, − 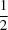 + *z*

**Table 3:** Percentage contributions of inter­atomic contacts to the Hirshfeld surface for (I)

Contact	Percentage contribution
H⋯H	50.2
C⋯H/H⋯C	31.6
O⋯H/H⋯O	10.7
N⋯H/H⋯N	4.2
N⋯O /O⋯N	0.9
C⋯O/O⋯C	0.9
C⋯C	0.8
C⋯N/N⋯C	0.7

**Table 4:** Experimental details

Crystal data	
Chemical formula	C_23_H_21_N_3_O_2_
*M*_r_	371.43
Crystal system, space group	Monoclinic, *P*2_1_/*n*
Temperature (K)	100
*a*, *b*, *c* (Å)	9.2879 (2), 21.9137 (3), 9.6740 (2)
β (°)	105.187 (2)
*V* (Å^3^)	1900.21 (6)
*Z*	4
Radiation type	Cu *K*α
μ (mm^−1^)	0.68
Crystal size (mm)	0.31 × 0.29 × 0.16

Data collection	
Diffractometer	Oxford Diffraction Xcaliber Eos Gemini
Absorption correction	Multi-scan (*CrysAlis PRO*; Agilent, 2011 ▸)
*T*_min_, *T*_max_	0.904, 1.000
No. of measured, independent and observed [*I* > 2σ(*I*)] reflections	25449, 3678, 3380
*R*_int_	0.025
(sin θ/λ)_max_ (Å^−1^)	0.615

Refinement	
*R*[*F* ^2^ > 2σ(*F* ^2^)], *wR*(*F* ^2^), *S*	0.038, 0.104, 1.03
No. of reflections	3678
No. of parameters	260
No. of restraints	2
H-atom treatment	H atoms treated by a mixture of independent and constrained refinement
Δρ_max_, Δρ_min_ (e Å^−3^)	0.22, −0.24

Computer programs: *CrysAlis PRO* (Agilent, 2011 ▸), *SHELXS* (Sheldrick, 2008 ▸), *SHELXL2014/7* (Sheldrick, 2015 ▸), *ORTEP-3 for Windows* (Farrugia, 2012 ▸), *DIAMOND* (Brandenburg, 2006 ▸) and *publCIF* (Westrip, 2010 ▸).

## supplementary crystallographic information

### Crystal data

**Table:**

C_23_H_21_N_3_O_2_	*F*(000) = 784
*M**_r_* = 371.43	*D*_x_ = 1.298 Mg m^−^^3^
Monoclinic, *P*2_1_/*n*	Cu *K*α radiation, λ = 1.5418 Å
*a* = 9.2879 (2) Å	Cell parameters from 12038 reflections
*b* = 21.9137 (3) Å	θ = 4.0–71.3°
*c* = 9.6740 (2) Å	µ = 0.68 mm^−^^1^
β = 105.187 (2)°	*T* = 100 K
*V* = 1900.21 (6) Å^3^	Slab (cut), light-yellow
*Z* = 4	0.31 × 0.29 × 0.16 mm

### Data collection

**Table:**

Oxford Diffraction Xcaliber Eos Gemini diffractometer	3678 independent reflections
Radiation source: fine-focus sealed tube	3380 reflections with *I* > 2σ(*I*)
Graphite monochromator	*R*_int_ = 0.025
Detector resolution: 16.1952 pixels mm^-1^	θ_max_ = 71.4°, θ_min_ = 4.0°
ω scans	*h* = −11→11
Absorption correction: multi-scan (CrysAlis PRO; Agilent, 2011)	*k* = −26→26
*T*_min_ = 0.904, *T*_max_ = 1.000	*l* = −11→11
25449 measured reflections	

### Refinement

**Table:**

Refinement on *F*^2^	Primary atom site location: structure-invariant direct methods
Least-squares matrix: full	Hydrogen site location: mixed
*R*[*F*^2^ > 2σ(*F*^2^)] = 0.038	H atoms treated by a mixture of independent and constrained refinement
*wR*(*F*^2^) = 0.104	*w* = 1/[σ^2^(*F*_o_^2^) + (0.0583*P*)^2^ + 0.6586*P*] where *P* = (*F*_o_^2^ + 2*F*_c_^2^)/3
*S* = 1.03	(Δ/σ)_max_ = 0.001
3678 reflections	Δρ_max_ = 0.22 e Å^−^^3^
260 parameters	Δρ_min_ = −0.24 e Å^−^^3^
2 restraints	

### Special details

**Table:**

Geometry. All esds (except the esd in the dihedral angle between two l.s. planes) are estimated using the full covariance matrix. The cell esds are taken into account individually in the estimation of esds in distances, angles and torsion angles; correlations between esds in cell parameters are only used when they are defined by crystal symmetry. An approximate (isotropic) treatment of cell esds is used for estimating esds involving l.s. planes.

### Fractional atomic coordinates and isotropic or equivalent isotropic displacement parameters (Å^2^)

**Table:**

	*x*	*y*	*z*	*U*_iso_*/*U*_eq_	
O1	1.01879 (9)	0.56831 (4)	0.59686 (10)	0.0282 (2)	
O18	0.65222 (10)	0.14747 (4)	0.28503 (10)	0.0303 (2)	
N1	0.81663 (12)	0.63230 (5)	0.54527 (11)	0.0253 (2)	
H1N	0.7293 (12)	0.6365 (7)	0.4858 (14)	0.030*	
N2	0.80445 (11)	0.53742 (5)	0.44127 (11)	0.0230 (2)	
H2N	0.8494 (16)	0.5033 (5)	0.4302 (16)	0.028*	
N3	0.65679 (11)	0.55029 (5)	0.37877 (11)	0.0224 (2)	
C1	0.88811 (13)	0.57941 (6)	0.53281 (13)	0.0227 (3)	
C2	0.86973 (13)	0.67870 (6)	0.64749 (13)	0.0234 (3)	
C3	0.94222 (14)	0.66456 (6)	0.78919 (14)	0.0267 (3)	
H3	0.9657	0.6234	0.8168	0.032*	
C4	0.97992 (14)	0.71113 (7)	0.88961 (14)	0.0314 (3)	
H4	1.0300	0.7017	0.9861	0.038*	
C5	0.94507 (14)	0.77139 (7)	0.85028 (15)	0.0337 (3)	
H5	0.9688	0.8029	0.9200	0.040*	
C6	0.87558 (14)	0.78537 (6)	0.70894 (16)	0.0312 (3)	
H6	0.8527	0.8266	0.6816	0.037*	
C7	0.83916 (13)	0.73922 (6)	0.60681 (14)	0.0263 (3)	
H7	0.7935	0.7490	0.5095	0.032*	
C8	0.57139 (13)	0.50863 (5)	0.30443 (12)	0.0215 (3)	
C9	0.61677 (13)	0.44598 (6)	0.28545 (13)	0.0221 (3)	
H9	0.7195	0.4382	0.2945	0.026*	
C10	0.52152 (13)	0.39879 (6)	0.25590 (12)	0.0223 (3)	
H10	0.4180	0.4081	0.2305	0.027*	
C11	0.56261 (13)	0.33419 (6)	0.25920 (12)	0.0216 (3)	
C12	0.45125 (13)	0.28994 (6)	0.21358 (13)	0.0239 (3)	
H12	0.3505	0.3026	0.1776	0.029*	
C13	0.48453 (14)	0.22853 (6)	0.21976 (13)	0.0253 (3)	
H13	0.4078	0.1994	0.1850	0.030*	
C14	0.63070 (14)	0.20925 (6)	0.27707 (13)	0.0232 (3)	
C15	0.74391 (14)	0.25212 (6)	0.32357 (13)	0.0253 (3)	
H15	0.8441	0.2392	0.3619	0.030*	
C16	0.70944 (14)	0.31380 (6)	0.31361 (13)	0.0247 (3)	
H16	0.7872	0.3429	0.3444	0.030*	
C17	0.78852 (18)	0.12581 (7)	0.37548 (15)	0.0384 (4)	
H17A	0.8711	0.1373	0.3351	0.058*	
H17B	0.7845	0.0813	0.3829	0.058*	
H17C	0.8041	0.1439	0.4709	0.058*	
C81	0.41306 (13)	0.52769 (5)	0.24448 (13)	0.0222 (3)	
C82	0.33269 (14)	0.55311 (6)	0.33371 (14)	0.0259 (3)	
H82	0.3795	0.5587	0.4327	0.031*	
C83	0.18453 (15)	0.57034 (6)	0.27873 (15)	0.0299 (3)	
H83	0.1298	0.5865	0.3408	0.036*	
C84	0.11639 (14)	0.56408 (6)	0.13362 (16)	0.0314 (3)	
H84	0.0157	0.5765	0.0960	0.038*	
C85	0.19547 (15)	0.53967 (7)	0.04391 (15)	0.0345 (3)	
H85	0.1494	0.5357	−0.0557	0.041*	
C86	0.34221 (15)	0.52092 (6)	0.09928 (14)	0.0298 (3)	
H86	0.3950	0.5033	0.0374	0.036*	

### Atomic displacement parameters (Å^2^)

**Table:**

	*U*^11^	*U*^22^	*U*^33^	*U*^12^	*U*^13^	*U*^23^
O1	0.0186 (4)	0.0265 (5)	0.0361 (5)	0.0021 (3)	0.0011 (4)	−0.0090 (4)
O18	0.0325 (5)	0.0216 (5)	0.0364 (5)	0.0031 (4)	0.0085 (4)	0.0038 (4)
N1	0.0203 (5)	0.0252 (5)	0.0256 (5)	0.0034 (4)	−0.0024 (4)	−0.0063 (4)
N2	0.0192 (5)	0.0194 (5)	0.0281 (5)	0.0018 (4)	0.0019 (4)	−0.0027 (4)
N3	0.0190 (5)	0.0224 (5)	0.0239 (5)	0.0002 (4)	0.0022 (4)	0.0004 (4)
C1	0.0200 (6)	0.0234 (6)	0.0241 (6)	−0.0006 (5)	0.0047 (5)	−0.0016 (5)
C2	0.0157 (5)	0.0270 (6)	0.0266 (6)	0.0003 (5)	0.0039 (5)	−0.0074 (5)
C3	0.0189 (6)	0.0330 (7)	0.0274 (6)	0.0020 (5)	0.0045 (5)	−0.0030 (5)
C4	0.0196 (6)	0.0488 (8)	0.0244 (6)	−0.0017 (6)	0.0035 (5)	−0.0097 (6)
C5	0.0218 (6)	0.0415 (8)	0.0379 (7)	−0.0047 (6)	0.0082 (5)	−0.0211 (6)
C6	0.0224 (6)	0.0273 (7)	0.0444 (8)	0.0006 (5)	0.0099 (6)	−0.0113 (6)
C7	0.0194 (6)	0.0286 (7)	0.0291 (6)	0.0025 (5)	0.0034 (5)	−0.0050 (5)
C8	0.0219 (6)	0.0215 (6)	0.0207 (6)	−0.0009 (5)	0.0046 (4)	0.0003 (4)
C9	0.0192 (6)	0.0236 (6)	0.0223 (6)	0.0013 (5)	0.0034 (4)	−0.0008 (4)
C10	0.0185 (6)	0.0254 (6)	0.0213 (6)	0.0010 (5)	0.0023 (4)	0.0000 (5)
C11	0.0212 (6)	0.0231 (6)	0.0200 (6)	−0.0004 (5)	0.0047 (4)	−0.0001 (4)
C12	0.0195 (6)	0.0256 (6)	0.0257 (6)	−0.0001 (5)	0.0044 (5)	0.0031 (5)
C13	0.0236 (6)	0.0237 (6)	0.0284 (6)	−0.0044 (5)	0.0066 (5)	0.0020 (5)
C14	0.0290 (6)	0.0210 (6)	0.0214 (6)	0.0016 (5)	0.0096 (5)	0.0022 (4)
C15	0.0214 (6)	0.0278 (6)	0.0256 (6)	0.0040 (5)	0.0041 (5)	−0.0014 (5)
C16	0.0211 (6)	0.0240 (6)	0.0278 (6)	−0.0024 (5)	0.0042 (5)	−0.0038 (5)
C17	0.0510 (9)	0.0276 (7)	0.0311 (7)	0.0153 (6)	0.0012 (6)	−0.0011 (6)
C81	0.0214 (6)	0.0155 (5)	0.0283 (6)	−0.0018 (4)	0.0036 (5)	0.0007 (5)
C82	0.0294 (7)	0.0212 (6)	0.0270 (6)	0.0012 (5)	0.0073 (5)	0.0036 (5)
C83	0.0305 (7)	0.0228 (6)	0.0400 (7)	0.0032 (5)	0.0159 (6)	0.0043 (5)
C84	0.0200 (6)	0.0262 (6)	0.0448 (8)	0.0016 (5)	0.0028 (6)	0.0028 (6)
C85	0.0278 (7)	0.0371 (8)	0.0321 (7)	0.0023 (6)	−0.0034 (5)	−0.0046 (6)
C86	0.0250 (7)	0.0329 (7)	0.0294 (7)	0.0023 (5)	0.0035 (5)	−0.0056 (5)

### Geometric parameters (Å, º)

**Table:**

O1—C1	1.2338 (15)	C10—H10	0.9500
O18—C14	1.3677 (15)	C11—C16	1.4002 (17)
O18—C17	1.4189 (17)	C11—C12	1.4020 (17)
N1—C1	1.3566 (16)	C12—C13	1.3787 (18)
N1—C2	1.4140 (16)	C12—H12	0.9500
N1—H1N	0.868 (9)	C13—C14	1.3909 (17)
N2—C1	1.3691 (16)	C13—H13	0.9500
N2—N3	1.3754 (14)	C14—C15	1.3937 (18)
N2—H2N	0.877 (9)	C15—C16	1.3865 (18)
N3—C8	1.2964 (16)	C15—H15	0.9500
C2—C7	1.3914 (18)	C16—H16	0.9500
C2—C3	1.3945 (18)	C17—H17A	0.9800
C3—C4	1.3891 (19)	C17—H17B	0.9800
C3—H3	0.9500	C17—H17C	0.9800
C4—C5	1.389 (2)	C81—C86	1.3943 (18)
C4—H4	0.9500	C81—C82	1.3964 (18)
C5—C6	1.385 (2)	C82—C83	1.3913 (19)
C5—H5	0.9500	C82—H82	0.9500
C6—C7	1.3923 (18)	C83—C84	1.387 (2)
C6—H6	0.9500	C83—H83	0.9500
C7—H7	0.9500	C84—C85	1.383 (2)
C8—C9	1.4616 (17)	C84—H84	0.9500
C8—C81	1.4922 (17)	C85—C86	1.3896 (19)
C9—C10	1.3422 (17)	C85—H85	0.9500
C9—H9	0.9500	C86—H86	0.9500
C10—C11	1.4643 (17)		
			
C14—O18—C17	117.36 (10)	C12—C11—C10	119.68 (11)
C1—N1—C2	125.97 (10)	C13—C12—C11	121.53 (11)
C1—N1—H1N	115.3 (11)	C13—C12—H12	119.2
C2—N1—H1N	118.7 (11)	C11—C12—H12	119.2
C1—N2—N3	118.57 (10)	C12—C13—C14	119.87 (11)
C1—N2—H2N	116.4 (10)	C12—C13—H13	120.1
N3—N2—H2N	125.0 (10)	C14—C13—H13	120.1
C8—N3—N2	119.61 (10)	O18—C14—C13	115.85 (11)
O1—C1—N1	124.31 (11)	O18—C14—C15	124.21 (11)
O1—C1—N2	120.62 (11)	C13—C14—C15	119.94 (11)
N1—C1—N2	115.07 (10)	C16—C15—C14	119.58 (11)
C7—C2—C3	120.00 (12)	C16—C15—H15	120.2
C7—C2—N1	118.66 (11)	C14—C15—H15	120.2
C3—C2—N1	121.19 (12)	C15—C16—C11	121.45 (12)
C4—C3—C2	119.51 (13)	C15—C16—H16	119.3
C4—C3—H3	120.2	C11—C16—H16	119.3
C2—C3—H3	120.2	O18—C17—H17A	109.5
C5—C4—C3	120.59 (13)	O18—C17—H17B	109.5
C5—C4—H4	119.7	H17A—C17—H17B	109.5
C3—C4—H4	119.7	O18—C17—H17C	109.5
C6—C5—C4	119.72 (12)	H17A—C17—H17C	109.5
C6—C5—H5	120.1	H17B—C17—H17C	109.5
C4—C5—H5	120.1	C86—C81—C82	118.53 (11)
C5—C6—C7	120.25 (13)	C86—C81—C8	121.24 (11)
C5—C6—H6	119.9	C82—C81—C8	120.23 (11)
C7—C6—H6	119.9	C83—C82—C81	120.48 (12)
C2—C7—C6	119.87 (12)	C83—C82—H82	119.8
C2—C7—H7	120.1	C81—C82—H82	119.8
C6—C7—H7	120.1	C84—C83—C82	120.22 (12)
N3—C8—C9	125.20 (11)	C84—C83—H83	119.9
N3—C8—C81	114.57 (11)	C82—C83—H83	119.9
C9—C8—C81	120.09 (10)	C85—C84—C83	119.78 (12)
C10—C9—C8	123.70 (11)	C85—C84—H84	120.1
C10—C9—H9	118.2	C83—C84—H84	120.1
C8—C9—H9	118.2	C84—C85—C86	120.07 (13)
C9—C10—C11	125.93 (11)	C84—C85—H85	120.0
C9—C10—H10	117.0	C86—C85—H85	120.0
C11—C10—H10	117.0	C85—C86—C81	120.88 (13)
C16—C11—C12	117.59 (11)	C85—C86—H86	119.6
C16—C11—C10	122.64 (11)	C81—C86—H86	119.6
			
C1—N2—N3—C8	−172.44 (11)	C10—C11—C12—C13	−177.65 (11)
C2—N1—C1—O1	−10.4 (2)	C11—C12—C13—C14	2.34 (19)
C2—N1—C1—N2	169.88 (12)	C17—O18—C14—C13	−163.67 (12)
N3—N2—C1—O1	176.21 (11)	C17—O18—C14—C15	15.55 (17)
N3—N2—C1—N1	−4.07 (16)	C12—C13—C14—O18	177.07 (11)
C1—N1—C2—C7	144.18 (13)	C12—C13—C14—C15	−2.19 (18)
C1—N1—C2—C3	−40.29 (19)	O18—C14—C15—C16	−178.53 (11)
C7—C2—C3—C4	1.68 (19)	C13—C14—C15—C16	0.66 (18)
N1—C2—C3—C4	−173.79 (11)	C14—C15—C16—C11	0.76 (19)
C2—C3—C4—C5	0.48 (19)	C12—C11—C16—C15	−0.63 (18)
C3—C4—C5—C6	−1.7 (2)	C10—C11—C16—C15	175.98 (12)
C4—C5—C6—C7	0.7 (2)	N3—C8—C81—C86	127.82 (13)
C3—C2—C7—C6	−2.62 (19)	C9—C8—C81—C86	−56.24 (16)
N1—C2—C7—C6	172.96 (11)	N3—C8—C81—C82	−51.92 (16)
C5—C6—C7—C2	1.42 (19)	C9—C8—C81—C82	124.02 (12)
N2—N3—C8—C9	3.18 (18)	C86—C81—C82—C83	0.97 (18)
N2—N3—C8—C81	178.88 (10)	C8—C81—C82—C83	−179.28 (11)
N3—C8—C9—C10	154.62 (12)	C81—C82—C83—C84	−1.90 (19)
C81—C8—C9—C10	−20.85 (18)	C82—C83—C84—C85	1.1 (2)
C8—C9—C10—C11	−169.19 (11)	C83—C84—C85—C86	0.6 (2)
C9—C10—C11—C16	9.05 (19)	C84—C85—C86—C81	−1.6 (2)
C9—C10—C11—C12	−174.41 (12)	C82—C81—C86—C85	0.76 (19)
C16—C11—C12—C13	−0.93 (18)	C8—C81—C86—C85	−178.98 (12)

### Hydrogen-bond geometry (Å, º)

*Cg*1 and *Cg*2 are the centroids of the C2–C7 and C81–C86 rings, respectively.

**Table:**

*D*—H···*A*	*D*—H	H···*A*	*D*···*A*	*D*—H···*A*
N1—H1*N*···N3	0.87 (1)	2.18 (2)	2.6029 (15)	110 (1)
N2—H2*N*···O1^i^	0.88 (1)	2.05 (1)	2.9184 (14)	171 (1)
C9—H9···O1^i^	0.95	2.39	3.2913 (15)	159
C15—H15···*Cg*1^i^	0.95	2.88	3.5125 (14)	125
C6—H6···*Cg*2^ii^	0.95	2.92	3.8296 (14)	161
C12—H12···*Cg*1^iii^	0.95	2.75	3.4715 (14)	133

Symmetry codes: (i) −*x*+2, −*y*+1, −*z*+1; (ii) *x*−1/2, −*y*+1/2, *z*−1/2; (iii) −*x*+1, −*y*+1, −*z*+1.
